# Disclosure experience and associated factors among HIV positive men and women clinical service users in southwest Ethiopia

**DOI:** 10.1186/1471-2458-8-81

**Published:** 2008-02-29

**Authors:** Kebede Deribe, Kifle Woldemichael, Mekitie Wondafrash, Amaha Haile, Alemayehu Amberbir

**Affiliations:** 1 Fayyaa Integrated Development Association-NCMI, PEPFAR-New Partners Initiative, P.O. Box 5035, Jimma, Ethiopia; 2 Jimma University, public health faculty, Department of Epidemiology and Biostatistics, Ethiopia; 3 Jimma University, public health faculty, Department of population and family health, Ethiopia; 4 Pre Service Education, JHPIEGO-Ethiopia, Addis Ababa, Ethiopia

## Abstract

**Background:**

Disclosing HIV test results to one's sexual partner allows the partner to engage in preventive behaviors as well as the access of necessary support for coping with serostatus or illness. It may motivate partners to seek testing or change behavior, and ultimately decrease the transmission of HIV. The present study was undertaken to determine the rate, outcomes and factors associated with HIV positive status disclosure in Southwest Ethiopia among HIV positive service users.

**Methods:**

A cross-sectional study was carried out from January 15, 2007 to March 15, 2007 in Jimma University Specialized Hospital. Data were collected using a pre-tested interviewer-administered structured questionnaire.

**Results:**

A total of 705 people (353 women and 352 men), participated in the study of which 71.6% were taking ART. The vast majority (94.5%) disclosed their result to at least one person and 90.8% disclosed to their current main partner. However, 14.2% of disclosure was delayed and 20.6% did not know their partner's HIV status. Among those who did not disclose, 54% stated their reason as fear of negative reaction from their partner. Among those disclosures however, only 5% reported any negative reaction from the partner. Most (80.3%) reported that their partners reacted supportively to disclosure of HIV status. Disclosure of HIV results to a sexual partner was associated with knowing the partner's HIV status, advanced disease stage, low negative self-image, residing in the same house with partner, and discussion about HIV testing prior to seeking services.

**Conclusion:**

Although the majority of participants disclosed their test results, lack of disclosure by a minority resulted in a limited ability to engage in preventive behaviors and to access support. In addition, a considerable proportion of the participants did not know their partner's HIV status. Programmatic and counseling efforts should focus on mutual disclosure of HIV test results, by encouraging individuals to ask their partner's HIV status in addition to disclosing their own.

## Background

The HIV/AIDS epidemic in Ethiopia continues to pose a threat to the lives of its people. It is estimated that 977,394 people live with the virus resulting in 71,902 HIV related deaths in 2007[1]. The national prevalence of HIV in 2007 is estimated to be 2.1% [1]. According to the Ethiopian Demographic and Health Survey (DHS) 2005 the prevalence of HIV among couples is 2.1% and the majorities (85.7%) of those are discordant. In addition most of these couples do not mutually know their HIV status [2]. Though the level of stigma is not well studied in the country, among DHS 2005 participants 65% of women and 77% of men say that they would not want to keep secret that a family member was infected with the AIDS virus and 59% of women and 72% of men say they would be willing to care for a family member with AIDS in their home [2].

The prevention and control of HIV infection depends on the prevention of new infections and treating currently infected individuals. Voluntary Counseling and Testing (VCT) is an entry point to prevention and treatment services [3,4]. VCT services place emphasis on HIV status disclosure among HIV-infected clients, particularly to their sexual partners. Many international organizations including UNAIDS, WHO and CDC emphasize the importance of HIV status disclosure [5,6].

Self-disclosure of sensitive information is generally thought to have beneficial effects on an individual's health, lower stress, and lead to better psychological health [7]. In the case of HIV/AIDS, individuals who disclose their status are in a better position in terms of reproductive choices as well as psychosocial support. In addition, disclosure facilitates other behaviors that may improve the management of HIV. For example, women who disclose their status to their partners may be more likely to participate in Prevention of Mother To Child Transmission (PMTCT) programs [8,9]. Studies also indicate that individuals who disclosed their results have better adherence to ART treatments [10]. A recent mathematical modeling analysis showed that serostatus disclosure reduced the risk of HIV transmission by 17.9% to 40.6% relative to non-disclosure. Increasing the disclosure rate from the base-case value of 51.9–75.7% produced a 26.2–59.2% reduction in risk [11].

The exchange of information about one's HIV status with a prospective partner is associated with safer sexual practices: when HIV-negative individuals are informed of a sexual partner's HIV infection, this information influences the types of sexual practices in which they choose to engage [12]. For instance among Ethiopian DHS-2005 respondents, 85% of women and 89% of men believe that, if a woman knows her husband has an STI, she is justified in either refusing to have sex with him or asking him to wear a condom [2]. However, disclosure does not always mean, individuals will use the information to protect themselves or others; in fact, some will knowingly place themselves at risk of infection [13]. Other studies have found no association between disclosure and safer sex [14,15].

Although disclosure has a number of benefits, it is not without problems. Along with the aforementioned benefits, HIV status disclosure has many potential risks and there are a number of barriers that HIV-infected individuals face when sharing their test results with friends, family and sexual partners. Results from different studies indicate that in the majority of the cases, support and understanding are the outcomes from partners upon disclosure of HIV test results [16-18]. However, disclosure can also incur negative consequences [18,19], making the decision to disclose a dilemma for individuals infected with HIV. The rate of HIV disclosure varies across different studies ranges 27% – 69% [17,19-23].

### Factors associated with HIV positive status disclosure

Numerous factors have been associated with status disclosure. Research on partnership variables has demonstrated that "main/steady/close/regular" partnerships are more likely to involve disclosure than "other/casual/unfamiliar" partnerships [24,25]. Factors beyond partnership can influence disclosure as well. Specifically, illness severity and length of time since HIV diagnosis have been shown to be positively correlated to disclosure [26]. HIV-infected individuals are more likely to disclose to a partner whom they know is HIV-positive than to an HIV-negative or unknown serostatus partner [25,27-29]. In a study conducted in Tanzania, a short duration of relationship, polygamous marriage, working out of home, not knowing someone with HIV and lower income were negatively associated with disclosure [30]. In the same study it was found that women who had greater than 6-lifetime sexual partners were less likely to disclose their status. Another factor associated with disclosure was social support. Individuals with high social support tend to disclose their result more often than those without such support [22]. Self-efficacy has been reported as one of the determinants of HIV status disclosure [20]. Having not disclosed to sex partners was closely associated with lower self-efficacy for disclosing, with women who had not disclosed reporting the lowest disclosure self-efficacy. In a study conducted in Tanzania [16] a very strong association was found between prior communication about HIV testing with a partner and HIV-serostatus disclosure. A similar finding was obtained from an Ethiopian study [17].

One study [31] found that women with higher education are more likely to disclose their result to their sexual partner than women who are illiterate. Gender is also found to be one of the associated factors of HIV status disclosure. In a study conducted in South Africa males were found to disclose their result more often to partner than females [32]. In contrast, another South African study [33] revealed that male sex is associated with non-disclosure of HIV status.

The aim of this study was to assess the magnitude of HIV status disclosure and non-disclosure, anticipated and actual outcomes of disclosure, and barriers and factors associated with HIV status disclosure. The findings from this study would help to understand the magnitude of HIV positive status disclosure, and provide evidence for HIV/AIDS prevention interventions in the country.

## Methods

### Study Setting

This study was conducted in Jimma University Specialized Hospital (JUSH). JUSH is found in Jimma Zone Southwest Ethiopia. The zone is one of 14 zones in Oromia Regional State; the capital Jimma, is located 335 KM Southwest of Addis Ababa. According to the AIDS in Ethiopia Sixth Report, HIV prevalence among pregnant women in the zone is 8.3% [1]. In the hospital, VCT, PMTCT, ART and treatment of opportunistic infection services are available. On record review as of December 25, 2006, 2036 PLWHA were found to be receiving services on an ongoing basis in the hospital. Data was collected from January 15, 2007 to March 15, 2007.

### Participants

The sample size was calculated using Epi-Info version 6.0 statistical software. Since one of the factors associated with disclosure to sexual partner is gender of the respondents, the result of a previous study [17] in Southwest Ethiopia that showed disclosure among women to be 69% was used. To detect a 10% difference in the rate of disclosure with 95% Confidence Interval (CI) and 80% power, a sample size of 331 in each group, male and female was calculated. With the addition of a 10% non-response rate, the final sample size became 706.

Of the 2036 PLWHA utilizing various ongoing services in the hospital, 915 were taking ART while the others were using pre-ART services. The 144 PLWHA aged less than 18 years were excluded from the preliminary survey. During the preliminary survey, 1576 (83.3%) PLWHA came to the hospital to receive services and were asked whether they currently have a sexual partner and are sexually active. A total of 856 PLWHA reported having a sexual partner and being sexually active. Out of 856 PLWHA eligible for the study we selected 706 randomly. The study was based on a sample of 706 randomly selected individuals (353 men and 353 women).

### Measurements

The dependent variable for this study was HIV positive status disclosure to a partner, recorded as yes or no.

The independent variables include socio-demographic characteristics (age, sex, income, education, religion, marital status, occupation, place of residence), relationship factors (number of partner, duration of relationship, type of partner, quality of relationship, discussion about HIV, fear of partner's reaction, HIV status of partner), illness related factors (stage of disease, duration of test result), service related factors(type of counseling, availability of follow up and ART), psycho-social factors (stigma, social support, support group membership, depression, active substance and alcohol use), and behavioral factors (self-efficacy, perceived severity, perceived benefit and perceived susceptibility).

Data were collected by a pre-tested questionnaire which was adopted from different studies [17,34,35]. The questionnaire includes demographic variables, health status variables, risk behaviors, partnership, partner characteristics, disclosure status, disclosure barriers, disclosure outcomes, social support and self-efficacy of disclosure. Perceived stigma was measured using 24 variables and depression was measured by BDI-13 to avoid the effects of overlapping symptoms of HIV disease and somatic depression that can inflate scores on the Beck Depression Inventory [36]. Health status variables included AIDS diagnosis based on WHO classification [37].

Single multiple response choice questions were asked to determine patterns of disclosure: "Have you told any one of the following that you are HIV-positive?" Then the outcomes of disclosure specific to sexual partner were assessed. If an individual did not disclose to their sexual partner, the reasons for non-disclosure were probed. To assess full disclosure versus, delayed disclosure the respondents were asked the following question "After you found out that you were infected, did you have sex with your partner before telling him/her about your infection?" Partnership characteristics included duration of the relationship, stability of the relationship, and partner HIV status as reported by participants (HIV-positive, HIV-negative or unknown), and the partners' education, income, and profession.

Stigma was measured using measures drawn from a previous scale on stigma previously used among people living with HIV/AIDS [38]. A set of 23 Likert scale questions addressing the perception of stigma and HIV were grouped into a composite index. Overall 23 questions were asked to measure stigma which was subdivided in to 10 questions for negative self image, 10 questions for negative public attitude and 7 questions for disclosure related stress. An example of one such question for negative self image is "I feel I am not as good a person as others because I have HIV;" for public attitude, "I worry that people may judge me when they learn I have HIV;" for disclosure stress, "In many areas of my life, no one knows that I have HIV." Response categories ranged from 1 to 4, for strongly disagree to strongly agree. The composite index was calculated as the mean of the Likert scale questions under each category combined, dichotomized into a variable indicating perception that HIV causes them stigma versus perception that there is limited stigma associated with their HIV status.

In this study casual partner refers to a sexual partner who is not a spouse. Partners with whom the individual had penetrative sexual intercourse with or with out consent. These include those individuals with whom he/she had once or few times sexual intercourse, other than regular partners and primary/regular partner refers to a sexual partner either spouse or one who lives together in the same house with in the 3 months prior to survey or partner with whom the respondent had regular sexual relationship and perceived by the respondent as regular boyfriend/girl friend.

Trained data collectors explained the aim of the study, obtained informed consent, and interviewed each respondent privately.

### Data analysis

Data were edited, cleaned, coded, entered and analyzed using SPSS version-12.0.1 for Windows. Descriptive statistics were calculated to determine rate of disclosure and other outcomes. Bivariate analyses were done to determine the presence of a statistically significant association between explanatory variables and the outcome variable. To identify independent associated factors multiple logistic regression were employed.

A logistic regression model was produced with disclosure and non-disclosure as outcome variable identifies associated factors. All explanatory variables that were associated with the outcome variable in bivariate analyses, variables with a P-value of ≤0.2 and variables consistently found to be associated with disclosure in other studies were included in the logistic models. P-value 0.2 was used to enter a variable in the model and 0.1 to remove a variable from the model.

### Human subjects

The study was approved by Jimma University Research Ethical Committee.

## Results

### Socio-demographic characteristics of the participants

A total of 705 (353 women and 352 men) respondents were interviewed yielding a response rate of 99.9%. Among the 705 subjects interviewed, the majority (50.8%) were between 26 and 35 years old, 396 (56.2%) had secondary educational status or higher, 341(48.4%) were Oromo by ethnicity, 525 (74.5%) resided in urban areas, 391 (55.6%) were employed and 418 (59.3%) were Orthodox Christians by religion. The median monthly family income was 250 Ethiopian Birr. The socio-demographic characteristics of the participants are summarized in Table 1.

**Table 1:** Basic socio-demographic characteristics of the respondents, Jimma University Specialized Hospital, March 2007

**Variable**	**Number**	**Percent**
Sex		
Male	352	49.9
Female	353	50.1
Age (years)		
< 20	32	4.5
21–25	134	19.0
26–35	358	50.8
> 36	181	25.7
Education		
Illiterate	101	14.3
Primary	208	29.5
Secondary	237	33.6
Post secondary	159	22.6
Ethnicity		
Oromo	341	48.4
Amhara	245	34.8
Dawro	51	7.2
Keffa	30	4.3
Tigre	15	2.1
Guraghe	11	1.6
Others	12	1.7
Religion		
Orthodox	418	59.3
Muslim	200	28.4
Protestant	81	11.5
Catholic	6	0.9
Place of residence		
Urban	525	74.5
Rural	180	25.5
Employment		
Employed	391	55.6
Not employed	312	44.4
Marital status		
Married	610	86.5
Unmarried	95	13.5
Family income ETB^a^		
<500	524	74.3
501–999	86	12.2
>1000	49	7.0
Unstated	46	6.5

a Exchange rate 1 USD = 8.6 Ethiopian Birr (ETB)

Among the 705 subjects interviewed, the majority (71.6%) were taking ART, and most (79.4%) of the participants knew their partner's HIV status. Most (67.5%) had HIV-positive partners, while 84 (11.9%) participants reported having HIV-negative partners and 145 (20.6%) had sexual partners whose HIV status they did not know. [See Table 2]

**Table 2:** Psychosocial, medical and sexual characteristics of the respondents, Jimma University Specialized Hospital, March 2007

**Variables**	**Male(352)**		**Female(353)**		**P-value**
	**N**	**%**	**N**	**%**	

Depression(BDI > 10)^a^					0.009
Not depressed	235	66.8	268	75.9	
Depressed	117	33.2	85	24.1	
Knowledge of partner's HIV status					0.468
Yes	284	80.7	276	78.2	
No	68	19.3	77	21.8	
Partner HIV status					0.517
HIV positive	238	67.6	238	67.4	
HIV negative	46	13.1	38	10.8	
Unknown	68	19.3	77	21.8	
Taking ART					0.000
yes	277	78.7	228	64.4	
No	75	21.3	125	35.4	
Number of life time Sexual partners					0.000
1	12	3.4	39	11.0	
2–4	182	51.7	241	68.3	
>= 5	158	44.9	73	20.7	
WHO Stage of disease at enrollment					
I & II	66	18.9	105	29.8	0.000
III	169	48.3	179	50.9	
IV	115	32.9	68	19.3	
Substance use ^b^					0.039
Yes	37	10.5	21	5.9	
No	315	89.5	332	94.1	

a Beck Depression Inventory

b Khat, Cigarettes, Alcohol.

### HIV status disclosure and sexual behaviors

Among the 705 participants, 666 (94.5%) indicated that they have disclosed their result to at least one individual and 640 (90.2%) respondents disclosed their result to their current main partner. However, of those who disclosed 91 (14.2%) had had sex with their partner before telling their result to their partner. Of these sexual encounters 63 (69.2%), 14 (15.4%) and 14 (15.4%) occurred with HIV positive, HIV negative and unknown HIV status partners, respectively. Only 5 (38.5%) of the 13 respondents who had a casual sexual partners reported disclosure to any of these partners as presented in Table 3.

**Table 3:** Rate of HIV status disclosure among HIV positive service users, Jimma University Specialized Hospital, March 2007

**Variables**	**Number**	**Percent**
Disclosure to anyone (n = 705)		
Yes	666	94.5
No	39	5.5
Disclosure to main sexual partner (n = 705)		
Yes	640	90.8
No	65	9.2
Disclosure to casual sexual partner (n = 13)		
Yes	5	38.5
No	8	61.5
Sex before disclosure (delayed disclosure) (n = 639)		
Yes	91	14.2
No	548	85.8
Knowledge of partner's HIV status (n = 705)		
Yes	560	79.4
No	145	20.6

Respondents reported disclosing most frequently to main partners (90.8%) followed by relatives (33.2%), mother (14.9%), friends (14.2%), father (9.1%), neighbors (6.8%), children (6%), other family members (4.7%) and religious leaders (4.4%).

Disclosure was made as early as one day and late as two years after learning serostatus. Most (73%) of the participants disclosed on the day of receiving test result, 74 (12%) within two weeks, 55 (9%) in 2 to 4 weeks, 27 (4%) in 1 to 4 months and 12 (2%) greater than 4 months.

### Reasons for non-disclosure

Reasons for non-disclosure among those respondents who did not disclose their test results to their partner (n = 65) were "my partner might get angry with me" (20.4%), "fear of separation/divorce" (17.3%), "my partner might be afraid of catching HIV from me" (16.3%), "not to worry partner"(9.2%), "fear of accusation of infidelity" (7.1%), "fear of being labeled a bad person" (6.1%), "no enough time to discuss because my partner works in other place" (6.1%), "fear of physical abuse" (5.1%), "fear of murder" (4.1%), "fear of breach of confidentiality" (3.1%) and other reasons presented in Table 4.

**Table 4:** Reasons for not disclosing HIV status to main sexual partner among HIV positive service users, Jimma University Specialized Hospital, March 2007

**Reasons for non-disclosure**	**Frequency**	**Percent**
He/She might get angry with me	20	20.4
He/She might leave me	17	17.3
He/She might be afraid of catching HIV from me	16	16.3
I do not want him/her to worry	9	9.2
He/She might think I am unfaithful	7	7.1
He/She might think I am a bad person	6	6.1
No enough time to discuss because he/she works far away	6	6.1
He/She might hurt me physically	5	5.1
He/She might kill me	4	4.1
He/She may tell others	3	3.1
There is no need to tell until I am sick	3	3.1
He/She has too many other problems to deal with at that time	1	1.0
He/She is too young to handle it	1	1.0

Among disclosures we were able to identify anticipated and actual outcomes to disclosure. As depicted in Table 5, 41.9% anticipated that their partner would be supportive while 46.4% of partners were supportive after disclosure. Moreover 27.7% anticipated their partner would assure them and in actual terms 38.4% received assurance from their partners. However, some of the partners reacted with unwanted reaction such as anger 24(2.4%), yelling 10 (1%) and rejection 1(0.1%). Even though there were participants who anticipated physical violence, no individuals were physically harmed.

**Table 5:** Anticipated versus actual main partner's reaction towards HIV status disclosure among HIV positive service users, Jimma University Specialized Hospital, March 2007

**Partner's reaction**	**Anticipated**	**Actual**
	N (%)	N (%)
Supportive	387(41.9)	470(46.4)
Reassuring	256(27.7)	389(38.4)
Annoyed	114(12.4)	24(2.4)
Confused	84(9.1)	54(5.3)
Cries	49(5.3)	55(5.4)
Talks about leaving the relationship	11(1.2)	1(0.1)
Worries about his/her own HIV status	6(0.7)	6(0.6)
Leaves the relationship	6(0.7)	1(0.1)
Asks about my sexual history	5(0.5)	2(0.2)
Threatens me	3(0.3)	0(0.0)
Beats me up	1(0.1)	0(0.0)
Yells at me	1(0.1)	10(1.0)
Any positive reactions ^a^	459(65.1)	566(80.3)
Any negative reactions ^a^	127(18.1)	37(5.2)

*a *Denominator N = 705

There was high agreement observed between anticipated and actual positive outcome of disclosure. Of 459 individuals who anticipated supportive outcomes, 96.3% (442/459) received support and assurance from their partner.

In contrast there was no agreement between anticipated and actual negative outcomes of disclosure. Of 127 respondents who anticipated a negative reaction from their partners, only 13.4% (17/127) faced adverse outcomes of disclosure; the rest (86.6%) received support and understanding from their partner. There was no significant difference in anticipated or actual outcome of HIV status disclosure between men and women.

Some (40%) of the participants identified someone to whom they did not want to tell their HIV positive status. Fifty-four percent said it was a neighbor, 20% a relative, 15% a partner, 3.9% a friend, 2.9% a workmate, 0.36% an employer and 3.2% someone else. The reasons that these individuals did not want to tell their HIV positive status were fear of stigma and discrimination (79.4%), not to worry others (13.8%), fear of gossip (6.5%) fear of a negative reaction (3.3%) and fear of losing their job (1.5%).

### Factors Associated with disclosure

In the multiple logistic regression model, five variables were found to be independently associated factors of disclosure to a partner. Individuals who live in the same house with their partner were 9.2 times more likely to disclose to their partner compared to those who do not live in the same house (OR, 9.25; 95% CI, 3.42–29.20). Nearly four times as many respondents who reported prior discussion about HIV testing disclosed to their partners in compared to those who reported not having a prior discussion about HIV (OR, 3.8; 95% CI, 1.6–8.6).

Knowledge of the HIV status of one's partner was also associated with, partner disclosure. Respondents that reported not knowing their partner's HIV status were 98% less likely to disclose to a partner in comparison with those who did know their partner's status (OR, 0.02; 95% CI, 0.01–0.04). Level of negative self-image was also another factor independently associated with disclosure. Respondents with a higher level of negative self-image were 97% less likely to disclose their result compared to those with a low level of negative self-image (OR, 0.03; 95% CI, 0.04–0.70).

The other factor associated with HIV status disclosure was clinical stage of disease. Individuals in an early clinical state (Stage I&II) of the WHO stage of disease were 78% less likely to disclose to a partner compared to those in an advanced (Stages III& IV) state of disease (OR, 0.22;95% CI, 0.10–0.55) as shown in Table 6.

**Table 6:** Factors independently associated with disclosure of HIV-positive test result to a partner among HIV-positive service users, Jimma University Specialized Hospital, March 2007

**Variables**	**Disclosed N (%)**	**Not disclosed N (%)**	**Crude OR(95% CI)**	**Adjusted^a ^OR (95% CI)**
Talked about testing with partner before test				
Yes	546(96.0)	23(4.0)	10.6(6.1–18.4)	3.8(1.6–8.6)^b^
No	94(69.1)	42(30.9)	1.0	1.0
Knowledge of partner's HIV status				
Yes	556(99.3)	4(0.7)	1.0	1.0
No	84(57.9)	61(42.1)	0.01(0.00–0.03)	0.02(0.01–0.04)^b^
WHO stage of disease				
Stage I-II	141(82.5)	30(17.5)	0.3(0.2–0.6)	0.22(0.10–0.55)^b^
Stage III – IV	496(93.4)	35(6.6)	1.0	1.0
Residing in the same house with partner				
Yes	544(93.8)	36(6.2)	4.6(2.7,7.8)	9.25(3.42–29.20)^b^
No	96(76.8)	29(23.2)	1.0	1.0
Negative self image				
Low	465(94.7)	26(5.3)	1.0	1.0
High	175(81.8)	39(18.2)	0.25(0.15–0.42)	0.03(0.04–0.70)^b^

a Adjusted for socio-demographic variables, psychosocial, partnership, illness and service related factors.

b P < 0.05 = statistically Significant

## Discussion

The current study focuses on determining rate, barriers and associated factors of HIV status disclosure to a sexual partner among service users in a hospital in Southwest Ethiopia. This study examined sexual activity before disclosure, approach that takes this factor into account may develop amore complete picture of disclosure than can be achieved by simply knowing the current disclosure status.

The general level of disclosure in this study was high: 94.5% disclosed to at least one person. This is comparable to other studies in Africa. In a study conducted in Uganda [39] it was found that 97% of subjects had disclosed their serostatus, two studies from South Africa ([2,40] found that more than 90% had disclosed their HIV serostatus to one or more people. However this is far higher than the one (60.5%) reported from Addis Ababa [23]. Concerning disclosure to one's partner, this study confirms that most participants have disclosed their HIV positive status to their sexual partner: only 9.2% of the people interviewed in this study did not disclose their status to their partner. This is higher than figures (7.6%) reported in Uganda [39] and lower than those found in South Africa (21%) and India (15%) [32,41]. The higher rate of disclosure in this study may be attributed to the presence of peer counselors which encourage disclosure and adherence to ART in the Hospital.

Despite the encouraging results found in this study, some (14.1%) of the disclosures were delayed, and these individuals had at least one sexual contact with their sexual partner before disclosure. This raises the possibility of transmission risk if condoms were not used [25] and may limit the beneficial aspect of disclosure, making negotiating safer sex difficult and perhaps putting the partner at risk of infection or re-infection. Our estimate of delayed disclosure is somewhat lower than that reported in another study [25]. Though most of the participants disclosed their result immediately, 27% concealed their HIV positive status for some time after learning their test results. This may lead to difficulty in negotiating safer sex and a risk reduction plans.

In contrary to what is reported in DHS Ethiopia 2005[2], in this study 67.4% reported an HIV positive partner and 78.2% reported knowing their partner's status. This discrepancy might be the result of difference in the study population. In the former case the study was community based and was conducted among individuals who may not know their result or those who deny their status. In the latter case the study was conducted in clinical set up among individuals who had accepted their result and who are taking services.

Consistent with other studies, the proportion of disclosure to main partners (90.8%) was found to be far greater than disclosure to casual partners (38.5%). One possible explanation for the low disclosure rate to casual partners is a difference in sense of responsibility. In one study, HIV positive individuals reported that they had a greater sense of responsibility to disclose toward partners with whom there was a shared emotional relationship, this sense of responsibility was lower or absent for non-primary partners [12]. These findings serve as a reminder that HIV-negative individuals should be educated about the need for consistent condom use with all sexual partners, particularly casual sex partners, and should not assume that HIV seropositive partners will inform them of their serostatus [42].

Despite the high rate of HIV status disclosure, a significant proportion (20.6%) of the respondents did not know their partner's HIV status. The silence of the partners could be either acknowledging that he/she is already infected or the result of the emotional rejection of the partner. However, one cannot rule out the possibility of shared fatalism in which the uninfected partners passively gives up him/her to acquiring the infection.

The reasons given for nondisclosure were fear of partner reaction, not wanting to worry their partner, fear of accusation of infidelity, lack of time for discussion, and fear of breach of confidentiality. Some women respondents feared physical abuse and murder. However in this study no individual experienced physical abuse. Indeed, feelings of uncertainty about how partners would react to their status were mentioned by most of the men and women participants. This is in agreement with other studies [17,43-46].

In this analysis, it was observed that there was high agreement between anticipated and actual positive outcomes of disclosure. Of the 459 individuals who anticipated supportive outcome to disclosure 96.3% (442/459) received support and assurance from their partner. In contrast there was no agreement between anticipated and actual negative outcomes of disclosure. Of the 127 respondents who anticipated a negative partner reaction only 13.4% (17/127) faced adverse outcomes of disclosure and the rest (86.6%) received support and understanding from their partners. In addition, the most commonly cited reason for non-disclosure was fear of partner's reaction; yet, only 5% of participants reported any negative partner's reaction. The majority of the disclosures received support and understanding from their partners. This suggests that HIV positive individuals tend to overestimate negative outcomes. Similar results have been reported in other studies [16,17]. However one can not exclude telescoping bias: respondents may tend to remember and report the earliest events which may under estimate negative reactions.

The results of this study are in agreement with many others [25,27-29,47] in that knowing a partner's HIV status was found to be associated with the disclosure of one's own status to a partner. However, along with other research [48], this study showed that it is not only knowing HIV positive status that is associated with disclosure, but knowing negative status is significantly associated with HIV status disclosure as well. This indicates that what matters is not the HIV status of the partner but rather knowledge of his/her status. It can also be implied that individuals who know their partner's HIV status have at least had a discussion about HIV testing; this would help them to anticipate their partner's reaction towards disclosure.

This study also suggests that many HIV-infected individuals delay disclosure until their disease has progressed. This might be because as the disease progresses, individuals find that they require emotional or material assistance from family, or it may simply reflect the fact that it becomes difficult to conceal their illness from their partners at a late disease stage. Other studies have found an association between disclosure and the experience of symptoms of AIDS [42,49,50]. Individuals living in the same house with their partner were more likely to disclose their result than those who do not live together. A similar finding was observed in Thailand [51].

The stigma related to HIV/AIDS and the behaviors associated with HIV risk have resulted in significant barriers to disclosing one's HIV infection status [20] to analyze the effect of stigma, we segregated measures for negative self-image, disclosure stress and perceived public attitude. Even though all were significantly associated with disclosure in bivariate analysis, in multivariate analysis only negative self image was found to be associated with HIV status disclosure. Supporting people to make effective decisions to disclose their HIV serostatus should be addressed in behavioral interventions like counseling on positive living and a multidisciplinary approach to develop positive self-image through follow up counseling.

Communicating with one's partner about testing prior to seeking service is significantly associated with disclosure. This might help individuals to anticipate a partner's reaction and would give them an opportunity to raise the issue again and disclose their result. Similar findings have been reported in other studies [16,17]. However, one study [30] did not find such an association.

The results of this study should be interpreted cautiously. First, the study was conducted among service users in a hospital setting. This setting may overestimate the disclosure rate and whose result cannot necessarily be generalized to other groups living with HIV/AIDS. Since participants were required to have a current partner to participate in the study the sample fails to capture those who had a partner, disclosed to the partner, and as a result ended their partnership prior to the interview. This may result in underestimate of negative outcomes of HIV status disclosure. The study was also limited in that it relied on self-report, and is therefore subject to reporting bias. The effect of social desirability bias and telescoping bias may be other potential limitations in this study. It would have been preferable to interview HIV-positive individuals who do not seek services, but considering ethical and practical issues, it was not possible in this study. With the aforementioned limitations in mind, we conclude that our findings have implications for interventions with PLWHA in the area.

## Conclusion

The results of this study suggest that HIV positive status disclosure among a segment of clinical service users is high. Although the findings may serve as the base-line data on the rate of HIV disclosure in the setup and thus assist in planning of HIV/AIDS prevention interventions, further data on HIV positive status disclosure with a relatively representative sample and prospective design will be needed to determine the rate, outcomes and determinants of HIV positive status disclosure in the study area.

Finally in Ethiopia where 85.7% of HIV positive couples are discordant and where most do not mutually know their HIV status [2], disclosure has paramount importance in curbing new infection. We suggest that HIV prevention in the country should target HIV status disclosure and further positive behavioral changes.

## Competing interests

The author(s) declare that they have no competing interests.

## Authors' contributions

KD conceived the study and analyzed and wrote the paper. KW, MW, AH and AA involved in designing the survey and undertook preliminary analysis. All contributed to the final report.

## Pre-publication history

The pre-publication history for this paper can be accessed here:



## References

[B1] Ministry Of Health Ethiopia & Federal HIV/AIDS Prevention and Control Office (2007). Single Point HIV prevalence estimate.

[B2] Central Statistical Agency and ORC Macro (2006). Ethiopia Demographic and Health Survey 2005.

[B3] Group VH-Ca TES (2000). Efficacy of voluntary HIV-1 counseling and testing in individuals and couples in Kenya, Tanzania and Trinidad: a randomized trial. The Lancet.

[B4] UNAIDS (2001). The impact of Voluntary Counseling and testing a global review of the benefits and challenges.

[B5] UNAIDS/WHO (2000). Opening up the HIV/AIDS Epidemic: Guidance on encouraging beneficial disclosure, ethical partner counseling & appropriate use of HIV case reporting.

[B6] CDC (2002). Revised guidelines for HIV counseling, testing and referral. MMWR Morbidity and Mortality Weekly Report.

[B7] Collins NL, Miller LC (1994). Self-Disclosure and Liking: A Meta-Analytic Review. Psychological Bulletin.

[B8] Medley A, Garcia-Morenoc C, MC Gill S, Maman S (2004). Rates, barriers and outcomes of HIV serostatus disclosure among women in developing countries; Implications for prevention of mother-to-child transmission programs. Bulletin of the World Health Organization.

[B9] WHO (2004). Gender Dimensions of HIV Status Disclosure to Sexual Partners: Rates, Barriers and Outcomes A Review Paper.

[B10] Waddell EN, Messeri PA (2006). Social support, disclosure, and use of antiretroviral therapy. AIDS Behav.

[B11] Pinkerton SD, Galletly CL (2007). Reducing HIV Transmission Risk by Increasing Serostatus Disclosure: A Mathematical Modeling Analysis. AIDS Behav.

[B12] Larkins S, Reback CJ, Shoptaw S, Veniegas R (2005). Methamphetamine-dependent gay men's disclosure of their HIV status to sexual partners. AIDS Care.

[B13] Serovich JM, Mosack KE (2003). Reasons for HIV disclosure or nondisclosure to casual sexual partners. AIDS Educ Prev.

[B14] Kalichman SC, Rompa D, Luke W, Austin J (2002). HIV transmission risk behaviors among HIV-positive persons in serodiscordant relationships. Int J STD AIDS.

[B15] Crepaz N, Marks G (2003). Serostatus disclosure, sexual communication and safer sex in HIV-positive men. AIDS Care.

[B16] Maman S, Mbwambo JK, Hogan NM, Weiss E, Kilonzo GP, Sweat MD (2003). High Rates and Positive Outcomes of HIV-Serostatus Disclosure to Sexual Partners: Reasons for Cautious Optimism from a Voluntary Counseling and Testing Clinic in Dar es Salaam, Tanzania. AIDS and Behav.

[B17] Kassaye KD, Lingerh W, Dejene Y (2005). Associated facors and outcomes of disclosing HIV-sero positive status to sexual partners among women in Mettu and Gore towns, Illubabor Zone southwest Ethiopia. Ethiop J Health Dev.

[B18] Gielen AC, O'campo P, Faden R, Eke A (1997). Women's disclosure of HIV status: experiences of mistreatment and violence in an urban setting. Women Health.

[B19] Temmerman M, Ndinya-Achola J, Ambani J, Piot P (1995). The right not to know HIV test results. The Lancet.

[B20] Kalichman SC, Nachimson D (1999). Self-Efficacy and Disclosure of HIV-Positive Serostatus to Sex Partners. Health Psychol.

[B21] Temmerman M, Moses S, Kiragu D, Fusallah S, Wamola IA, Piot P (1990). Impact of single session post-partum counseling of HIV infected women on their subsequent reproductive behavior. AIDS Care.

[B22] Degefa A, Sanders EJ, Mekonnen Y, Messele T, Wolday D, Dorigo-Zetsma W, Mekonnen W, Schaap A, Dukers NH (2003). Knowledge and attitude towards anti retroviral therapy among factory workers participating in a cohort on HIV and AIDS, Addis Ababa, Ethiopia. EMJ.

[B23] Tadios Y, Davey G (2006). Antiretroviral treatment adherence and its correlates in Addis Ababa, Ethiopia. Ethiop Med J.

[B24] Misovich SJ, Fisher JD, Fisher WA (1997). Close relationships and elevated HIV risk behavior: evidence and possible underlying psychological processes. Rev Gen Psychol.

[B25] Niccolai LM, King E, D'entremont D, Pritchett EN (2006). Disclosure of HIV Serostatus to Sex Partners: A New Approach to Measurement. Sexually Transmitted Diseases.

[B26] Mansergh G, Marks G, Simoni JM (1995). Self-disclosure of HIV infection among men who vary in time since seropositive diagnosis and symptomatic status. AIDS.

[B27] Marks G, Ruiz MS, Richardson JL, Reed D, Mason HR, Sotelo M, Turner PA (1994). Anal intercourse and disclosure of HIV infection among seropositive gay and bisexual men. J Acquir Immune Defic Syndr.

[B28] Marks G, Crepaz N (2001). HIV-positive men's sexual practices in the context of self-disclosure of HIV status. J Acquir Immune Defic Syndr.

[B29] Rosa CJ, Marks G (1998). Preventive counseling of HIV-positive men and self-disclosure of serostatus to sex partners: New opportunities for prevention. Health Psychol.

[B30] Antelman G, Fawzi S, Mary C, Sylvia K, Jessie M, Msamanga GI, Hunter DJ, Fawzi WW (2001). Associated factors of HIV-1 status disclosure: A prospective study among HIV-infected pregnant women in Dar es Salaam, Tanzania. AIDS.

[B31] Issiaka S, Cartoux M, Ky-Zerbo O, Tiendrebéogo S, Meda N, Dabis F, Van de Perre P, Ditrame Study Group (2001). Living with HIV: women's experience in Burkina Faso, West Africa. AIDS Care.

[B32] Skogmar S, Shakely D, lans M, danell J, Andersson R, Tshandu N, Ode'n A, Roberts S, Francois Venter WD (2006). Effect of antiretroviral treatment and counseling on disclosure of HIV-serostatus in Johannesburg, South Africa. AIDS Care.

[B33] Olley BO, Seedat S, Stein DJ (2004). Self-Disclosure of HIV Serostatus in Recently Diagnosed Patients with HIV in South Africa. African Journal of Reproductive Health.

[B34] Huba GJ, Melchior LA (1996). Staff of the Measurement Group, and the Washington University HRSA SPNS Cooperative Agreement Project. Module 75: DisclosureQuestionnaire.

[B35] Sauka M, Lie GT (2000). Confidentiality and disclosure of HIV infection: HIV-positive persons' experience with HIV testing and coping with HIV infection in Latvia. AIDS Care.

[B36] Kalichman SC, Rompa D, Cage M (2000). Distinguishing between Overlapping Somatic Symptoms of Depression and HIV Disease in People Living with HIV-AIDS. The Journal of Nervous and Mental Disease.

[B37] WHO (1990). AIDS interim proposal for a WHO staging system for HIV infection and Disease. Weekly Epidem Rec.

[B38] Berger BE, Ferrans CE, Lashley FR (2001). Measuring stigma in people with HIV: psychometric assessment of the HIV stigma scale. Res Nurs Health.

[B39] Nakayiwa S Disclosure Experiences Among HIV-Infected Persons Taking Anti-Retroviral Therapy in Rural Uganda [Abstract]. Abstract number. 509. The President's Emergency Plan for AIDS Relief Annual Meeting The 2006 HIV/AIDS Implementers' Meeting Building on Success: Ensuring Long-Term Solutions June 12–15, 2006, Durban, South Africa.

[B40] Nachega JB, Lehman DA (2005). HIV/AIDS and antiretroviral treatment knowledge, attitudes, beliefs, and practices in HIV-infected adults in Soweto, South Africa. J Acquir Immune Defic Syndr.

[B41] Chandra SP, Deepthivarma S, Manjula V (2003). Disclosure of HIV infection in South India patterns, reasons and reactions. AIDS Care.

[B42] O'Brien ME, Richardson-Alston G, Ayoub M, Magnus M, Peterman TA, Kissinger P (2003). Prevalence and correlates of HIV serostatus disclosure. SexTransm Dis.

[B43] Kalichman SC, Nachimson D, Cherry C, Williams E (1998). AIDS treatment advances and behavioral prevention setbacks: preliminary assessment of reduced perceived threat of HIV-AIDS. Health Psychol.

[B44] Chin D, Kroesen KW (1999). Disclosure of HIV infection among Asian/Pacific Islander American women: Cultural stigma and support. Cultural Diversity and Ethnic Minority Psychology.

[B45] Kilewo C, Massawe A, Lyamuya E, Semali I, Kalokola F, Urassa E, Giatas M, Temu F, Karlsson K, Mhalu F, Biberfeld G (2001). HIV counseling and testing of pregnant women in Sub-Saharan Africa. J Acquir Immune Defic Syndr.

[B46] Rakwar J, Kidula N, Fonck K, Kirui P, Ndinya-Achola J, Temmerman M (1999). HIV/STD: the women to blame? Knowledge and attitudes among STD clinic attendees in the second decade of HIV/AIDS. International Journal of STD & AIDS.

[B47] Simbayi LC, Kalichman SC, Strebel A, Cloete A, Henda N, Mqeketo A (2007). Disclosure of HIV status to sex partners and sexual risk behaviors among HIV-positive men and women, Cape Town, South Africa. Sex Transm Inf.

[B48] Dave SS, Stephenson J, Mercey ED, Panahmand N, Jungmann E (2006). Sexual behavior, condom use, and disclosure of HIV status in HIV infected heterosexual individuals attending an inner London HIV clinic. Sex Transm Inf.

[B49] Hays R, McKusick L, Pollack L, Hilliard R, Hoff C, Coates T (1993). Disclosing HIV seropositivity to significant others. AIDS.

[B50] Marks G, Bundek N, Richardson J, Ruiz M, Maldonado N, Mason H (1992). Self-disclosure of HIV infection: preliminary results from a sample of Hispanic men. Health Psychol.

[B51] Skunodom N, Linkins RW, Culnane ME, Prymanee J, Kannasoot C, Suwannapha W, Suwanmaitre S, Utenpitak C, Yuvasevee C, Teeraratkul A, Simonds RJ, Tappero JW (2006). Factors associated with non-disclosure of HIV infection status of new mothers in Bangkok. Southeast Asian J Trop Med Public Health.

